# Machine learning prediction and SHAP interpretability analysis of heart failure risk in patients with hyperuricemia

**DOI:** 10.3389/fcvm.2025.1689607

**Published:** 2025-12-01

**Authors:** Tian-ming Gan, Shi-rong Wang, Guan-lian Mo, Shu-hu Li, Yong-qi Lu, Jin-yi Li

**Affiliations:** Department of Cardiology, The First Affiliated Hospital of Guilin Medical University, Guilin, Guangxi, China

**Keywords:** hyperuricemia, heart failure, machine learning, SHAP, NHANES

## Abstract

**Aims:**

Cardiovascular disorders, particularly heart failure (HF), are a critical global health challenge. Hyperuricemia, a key cardiovascular risk factor, significantly increases HF susceptibility. Existing HF risk prediction tools are often cumbersome, relying on extensive clinical parameters and tests, limiting their practical use. Therefore, there is an urgent need for a simple, interpretable model to assess HF risk in hyperuricemia patients.

**Methods and results:**

Using 2005–March 2020 NHANES data (85,750 participants), 1,603 adults (≥18 years) with confirmed hyperuricemia were included. Accessible multidimensional indicators were selected for routine clinical use. Multiple machine learning models (Random Forest, Logistic Regression, XGBoost, SVM, etc.) were applied, with performance evaluated via accuracy, sensitivity, F1-score, and ROC AUC, etc. SHAP values analyzed feature importance for the best model. The SVM model showed the best overall performance, with chronic kidney disease, coronary heart disease, hypertension, serum potassium, serum osmolality, and sedentary time emerging as the top predictors. These six indicators demonstrated strong predictive power for HF in hyperuricemia patients, highlighting their clinical relevance.

**Conclusion:**

Integration of these six readily available indicators provides a simple, interpretable tool for HF risk stratification in hyperuricemia patients. While further longitudinal and multicenter validation is needed, the model shows promise for early identification and targeted intervention in clinical practice.

## Introduction

Heart failure (HF) is a prevalent and debilitating disease that imposes a substantial burden on global health. Current estimates indicate that HF affects approximately 1%–2% of the adult population worldwide. The incidence and prevalence of HF are on the rise, underscoring the importance of understanding its pathogenesis and management. HF is characterized by a complex interplay of multiple factors, and despite significant advances in treatment, its morbidity and mortality remain high (1–3).

Serum uric acid (SUA), the end product of purine metabolism, has emerged as a potential biomarker for HF and plays a crucial pathogenic role in heart failure (4–6). A considerable proportion of HF patients (approximately 20%–50%) are diagnosed with hyperuricemia (7, 8). Hyperuricemia, a common metabolic syndrome, is a recognized risk factor for HF, and elevated SUA concentrations are associated with poor prognosis in HF patients (9–11). However, the underlying pathogenic mechanisms of SUA in HF remain unclear. Increased oxidative stress, activation of proinflammatory pathways, and impaired fatty acid metabolism caused by elevated SUA levels may be potential molecular mechanisms (12–14).

Identifying factors influencing heart failure onset/progression in hyperuricemia patients is key for early management. Current HF risk tools, relying on complex parameters/tests, are limited in clinical use, necessitating a simple, reliable prediction model.

Machine learning (ML) can capture complex patterns and non-linear relationships in high-dimensional data—critical for multifactorial diseases like HF—outperforming traditional models in risk stratification (15, 16).

Using 2005–March 2020 NHANES data, this study will build an ML-based HF risk model for adults (≥18 years) with hyperuricemia, exploring multidimensional indicators' predictive value via multiple ML algorithms. The goal is a simple, high-performance tool to aid early identification of high-risk individuals and targeted interventions.

## Methods

### Data sources and study population

This study used public NHANES data—an ongoing, comprehensive cross-sectional initiative collecting interviews, physical exams, and lab data (available at https://www.cdc.gov/nchs/nhanes). Approved by the National Center for Health Statistics' ethics board, its health indicators are reliable.

Including 2005–March 2020 data (initial 85,750 participants), 1,603 adults (≥18 years) with hyperuricemia (after excluding incomplete data) were analyzed. Hyperuricemia was defined as a serum uric acid level ≥420 μmol/L in men and ≥360 μmol/L in women. Rigorously cleaned/preprocessed data trained/tested ML models to predict heart failure risk in these patients.

### Variable selection and determination

This study, through a literature review, selected multiple features from the NHANES database for analysis. The features chosen were as follows: (1) demographic factors, including gender, age, race, marital status, and educational attainment; (2) anthropometric measures, such as body mass index (BMI), height, and waist circumference; (3) lifestyle aspects, covering alcohol intake, sedentary habits, and smoking status; (4) biochemical test findings, comprising serum albumin, alanine aminotransferase, total calcium, total cholesterol, γ-glutamyl transferase, bicarbonate, blood glucose, iron, triglycerides, potassium, uric acid, sodium, osmolality, globulin, low-density lipoprotein (LDL) cholesterol, high-density lipoprotein (HDL) cholesterol, and glycosylated hemoglobin; (5) blood test results, involving lymphocyte count, monocyte count, neutrophil count, neutrophil proportion, and platelet count; (6) medical history, including diabetes, coronary heart disease (CHD/CAD: participants were categorized as having CHD/CAD when answering “yes” to the question “Has a doctor or other healthcare professional ever informed you that you have coronary heart disease/angina/heart attack (also known as myocardial infarction)?”), heart failure, hypertension, asthma, arthritis, cancer, hyperlipidemia (triglycerides ≥150 mg/dl, or total cholesterol ≥200 mg/dl, or LDL ≥130 mg/dl, or HDL-C <40 mg/dl in males, or HDL-C <50 mg/dl in females), and chronic kidney disease (eGFR <60 or ACR (urine albumin/creatinine ratio) >30); (7) composite inflammatory indices: NPAR, NLR, SII, and SIRI.

The rationale for including these candidate features was based on literature evidence and clinical availability. Features were chosen if they are closely associated with heart failure and cardiovascular risk, while features with limited clinical relevance or not routinely measured in practice were excluded.

The dependent variable (output variable) was defined as whether the participant had received a doctor - diagnosed heart failure. Based on this definition, participants were categorized into the heart failure group and the non - heart failure group.

### Data pre-processing and statistical analysis

Statistical analyses used R 4.3.1. Normally distributed continuous variables: mean ± SD with *t*-test for group comparisons. Non-normal ones: median (lower quartile, upper quartile) with non-parametric tests. Categorical variables: frequencies/percentages with χ^2^ test. Significance: *P* < 0.05.

Data preprocessing included normalization/standardization to boost model performance and consistency, improving dataset quality for reliable machine learning models.

Class imbalance in the dataset was addressed using the SMOTEENN algorithm, which increases minority samples while reducing majority samples to produce a balanced dataset. This approach ensures that the model can learn effectively from the limited number of HF events while reducing bias towards the majority class, enhancing model stability and predictive performance.

Feature selection was conducted using LASSO logistic regression on the scaled feature matrix from the balanced dataset. A 10-fold cross-validation procedure was applied to select features with non-zero coefficients for subsequent machine learning modeling. Prior to model training, collinearity among selected features was assessed to ensure model stability.

The dataset (7:3 train-test split) was used to develop/evaluate 8 ML models (logistic regression, kNN, decision tree, random forest, XGBoost, LightGBM, SVM, neural network). Performance was assessed via accuracy, sensitivity, specificity, precision, negative predictive value, F1, AUC (discrimination), Hosmer-Lemeshow test (calibration), and decision curves (clinical utility).

To further interpret the internal mechanism of the optimal machine learning model, SHAP (SHapley Additive exPlanations) analysis was applied to quantify and visualize the contribution of each feature to model predictions. SHAP values are derived from cooperative game theory and represent a fair allocation of the model's predicted outcome among all input features, treating each feature as a “player” participating in the final prediction. Following the properties of Shapley values—efficiency, symmetry, additivity, and the null player—SHAP provides an unbiased estimation of each feature's marginal impact on individual predictions. For a given participant, the SHAP value indicates the extent to which each feature shifts the predicted probability of heart failure relative to the model's average prediction, thereby elucidating the direction and magnitude of each feature's effect. The features selected through LASSO logistic regression were further examined using visual representations of their contributions and effects within the model, allowing the relationships between key predictors and heart failure risk to be clearly conveyed.

## Results

### Determination of study participants

**Figure 1:**
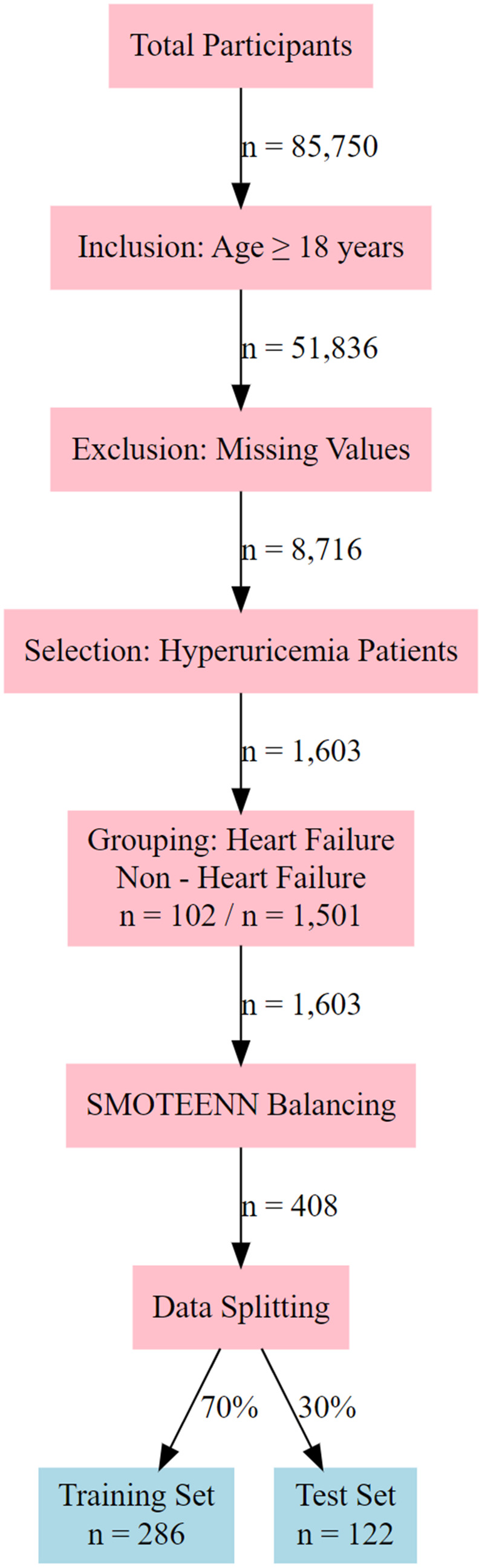
Flowchart of participant selection and data processing for hyperuricemia - heart failure study.

The study participants were identified through a systematic screening and processing procedure. Initially, 85,750 participants were included, among whom 51,836 met the age criterion (≥18 years). After excluding cases with missing data, 8,716 participants remained. From these, 1,603 patients with hyperuricemia were selected and divided into a heart failure group (*n* = 102) and a non-heart failure group (*n* = 1,501). The remaining dataset was subjected to imbalance correction using the SMOTEENN algorithm, resulting in 408 balanced samples. The final dataset was randomly split into a training set (*n* = 286) and a test set (*n* = 122) at a ratio of 70:30 for model performance evaluation (Figure 1). This process ensured the representativeness of the samples and statistical validity, with the sample size recorded at each stage to facilitate the reproducibility of the results.

### Characteristics of study variables and comparison between HF and non-HF groups

This study compared the clinical characteristics of 102 heart failure (HF) patients and 1,501 non-heart failure patients, with results showing significant differences between the two groups (Table 1).

**Table 1:** Characteristics of study variables and comparison between HF and Non-HF groups.

Variable	Overall (*N* = 1,603)	Non-heart failure (*N* = 1,501)	Heart failure (*N* = 102)	*P*-value
Categorical variable				
Coronary heart disease (%)	No: 1,431 (89.3%)	No: 1,389 (92.5%)	No: 42 (41.2%)	*p* < 0.001
	Yes: 172 (10.7%)	Yes: 112 (7.5%)	Yes: 60 (58.8%)	
Sex (%)	Male: 894 (55.8%)	Male: 843 (56.2%)	Male: 51 (50.0%)	*p* = 0.267
	Female: 709 (44.2%)	Female: 658 (43.8%)	Female: 51 (50.0%)	
Age (%)	18–59 years: 890 (55.5%)	18–59 years: 873 (58.2%)	18–59 years: 17 (16.7%)	*p* < 0.001
	≥60 years: 713 (44.5%)	≥60 years: 628 (41.8%)	≥60 years: 85 (83.3%)	
Race (%)	Mexican American: 180 (11.2%)	Mexican American: 172 (11.5%)	Mexican American: 8 (7.8%)	*p* = 0.254
	Non-Hispanic Black: 379 (23.6%)	Non-Hispanic Black: 350 (23.3%)	Non-Hispanic Black: 29 (28.4%)	
	Non-Hispanic White: 758 (47.3%)	Non-Hispanic White: 706 (47.0%)	Non-Hispanic White: 52 (51.0%)	
	Other: 286 (17.8%)	Other: 273 (18.2%)	Other: 13 (12.7%)	
Hypertension (%)	No: 724 (45.2%)	No: 710 (47.3%)	No: 14 (13.7%)	*p* < 0.001
	Yes: 879 (54.8%)	Yes: 791 (52.7%)	Yes: 88 (86.3%)	
Stroke (%)	No: 1,516 (94.6%)	No: 1,437 (95.7%)	No: 79 (77.5%)	*p* < 0.001
	Yes: 87 (5.4%)	Yes: 64 (4.3%)	Yes: 23 (22.5%)	
Diabetes mellitus (%)	No: 1,308 (81.6%)	No: 1,250 (83.3%)	No: 58 (56.9%)	*p* < 0.001
	Yes: 295 (18.4%)	Yes: 251 (16.7%)	Yes: 44 (43.1%)	
Asthma (%)	No: 1,348 (84.1%)	No: 1,269 (84.5%)	No: 79 (77.5%)	*p* = 0.079
	Yes: 255 (15.9%)	Yes: 232 (15.5%)	Yes: 23 (22.5%)	
Arthritis (%)	No: 1,074 (67.0%)	No: 1,025 (68.3%)	No: 49 (48.0%)	*p* < 0.001
	Yes: 529 (33.0%)	Yes: 476 (31.7%)	Yes: 53 (52.0%)	
Cancer (%)	No: 1,417 (88.4%)	No: 1,343 (89.5%)	No: 74 (72.5%)	*p* < 0.001
	Yes: 186 (11.6%)	Yes: 158 (10.5%)	Yes: 28 (27.5%)	
Marital status (%)	Never smoker/Living with partner: 959 (59.8%)	Never smoker/Living with partner: 911 (60.7%)	Never smoker/Living with partner: 48 (47.1%)	*p* < 0.001
	Widowed/Divorced/Separated: 405 (25.3%)	Widowed/Divorced/Separated: 355 (23.7%)	Widowed/Divorced/Separated: 50 (49.0%)	
	Never married: 239 (14.9%)	Never married: 235 (15.7%)	Never married: 4 (3.9%)	
Educational attainment (%)	<High school: 359 (22.4%)	<High school: 329 (21.9%)	<High school: 30 (29.4%)	*p* = 0.102
	≥High school: 1,244 (77.6%)	≥High school: 1,172 (78.1%)	≥High school: 72 (70.6%)	
Smoking history (%)	Never smoker: 821 (51.2%)	Never smoker: 774 (51.6%)	Never smoker: 47 (46.1%)	*p* = 0.514
	Former smoker: 492 (30.7%)	Former smoker: 456 (30.4%)	Former smoker: 36 (35.3%)	
	Current smoker: 290 (18.1%)	Current smoker: 271 (18.1%)	Current smoker: 19 (18.6%)	
BMI (%)	Normal weight: 216 (13.5%)	Normal weight: 202 (13.5%)	Normal weight: 14 (13.7%)	*p* = 0.929
	Underweight: 6 (0.4%)	Underweight: 6 (0.4%)	Underweight: 0 (0.0%)	
	Overweight: 500 (31.2%)	Overweight: 469 (31.2%)	Overweight: 31 (30.4%)	
	Obesity: 881 (55.0%)	Obesity: 824 (54.9%)	Obesity: 57 (55.9%)	
Alcohol drinking history (%)	No: 416 (26.0%)	No: 382 (25.4%)	No: 34 (33.3%)	*p* = 0.101
	Yes: 1,187 (74.0%)	Yes: 1,119 (74.6%)	Yes: 68 (66.7%)	
Hyperlipidemia (%)	No: 409 (25.5%)	No: 380 (25.3%)	No: 29 (28.4%)	*p* = 0.561
	Yes: 1,194 (74.5%)	Yes: 1,121 (74.7%)	Yes: 73 (71.6%)	
Chronic kidney disease (%)	No: 1,128 (70.4%)	No: 1,105 (73.6%)	No: 23 (22.5%)	*p* < 0.001
	Yes: 475 (29.6%)	Yes: 396 (26.4%)	Yes: 79 (77.5%)	
Continuous variables				
Fasting blood glucose (mg/dl)	100 [97, 117]	100 [97, 116]	110 [103.25, 130]	*p* < 0.001
Glycated hemoglobin (HbA1c, %)	5.7 [5.4, 6.1]	5.6 [5.3, 6.1]	6.1 [5.73, 6.7]	*p* < 0.001
Waist circumference (cm)	110 [96.4, 117.2]	110 [96.4, 117.1]	110 [100.17, 118.77]	*p* = 0.063
Height (cm)	170 [160.9, 176.3]	170 [161.1, 176.6]	170 [156.6, 172.57]	*p* = 0.001
Serum albumin (g/L)	42 [40, 45]	43 [40, 45]	41 [39, 43]	*p* < 0.001
Serum globulin (g/L)	30 [27, 33]	30 [27, 33]	30 [27, 34]	*p* = 0.495
Alanine transaminase (ALT, U/L)	24 [18, 32]	24 [18, 32]	20 [15.25, 26.75]	*p* = 0.001
Aspartate transaminase (AST, U/L)	25 [21, 30]	25 [21, 30]	24 [20, 30]	*p* = 0.502
Gamma-glutamyl transferase (GGT, IU/L)	25 [17, 38]	25 [17, 38]	26 [17, 44]	*p* = 0.492
Serum bicarbonate (mmol/L)	25 [23, 27]	25 [23, 26]	25 [24, 27]	*p* = 0.710
Serum osmolality (mmol/kg)	280 [276, 283]	280 [276, 282]	280 [280, 288]	*p* < 0.001
Serum total calcium (mmol/L)	2.4 [2.3, 2.4]	2.4 [2.3, 2.4]	2.3 [2.28, 2.4]	*p* = 0.041
Serum potassium (mmol/L)	4.0 [3.8, 4.3]	4.0 [3.8, 4.3]	4.2 [4.0, 4.46]	*p* < 0.001
Serum sodium (mmol/L)	140 [138, 141]	140 [138, 141]	140 [138, 142]	*p* = 0.034
Serum glucose (mmol/L)	5.5 [5.05, 6.16]	5.4 [5.0, 6.11]	6.2 [5.38, 6.94]	*p* < 0.001
Serum iron (μmol/L)	15 [11.5, 19.3]	15 [11.6, 19.5]	14 [11.1, 17.4]	*p* = 0.026
Sedentary time (minutes)	360 [240, 480]	360 [240, 480]	450 [240, 600]	*p* = 0.007
NPAR	140 [118.74, 152.86]	130 [118.14, 151.90]	150 [130.06, 167.29]	*p* < 0.001
SII	440,000 [319,328.74, 642,066.67]	440,000 [319,724.14, 635,444.44]	500,000 [316,228.57, 762,244.24]	*p* = 0.152
NLR	1.9 [1.43, 2.62]	1.9 [1.41, 2.54]	2.4 [1.66, 3.87]	*p* < 0.001
SIRI	1.0 [0.69, 1.51]	0.98 [0.68, 1.47]	1.5 [0.91, 2.33]	*p* < 0.001

Among categorical variables, 58.8% of the heart failure group had coronary heart disease, compared to only 7.5% in the non-heart failure group; 83.3% of the heart failure group were elderly patients, vs. 41.8% in the non-heart failure group; 86.3% of patients in the heart failure group had hypertension, compared with 52.7% in the non-heart failure group; 22.5% of heart failure patients had a history of stroke, vs. 4.3% in the non-heart failure group; 43.1% of heart failure patients had diabetes, while the proportion in the non-heart failure group was 16.7%; 52.0% of heart failure patients suffered from arthritis, compared with 31.7% in the non-heart failure group; 27.5% of heart failure patients had a history of cancer, vs. 10.5% in the non-heart failure group; 49.0% of heart failure patients had the marital status of “married and living with a partner”, compared with 23.7% in the non-heart failure group; and 77.5% of heart failure patients had chronic kidney disease (CKD), vs. 26.4% in the non-heart failure group. Variables not significantly associated with heart failure (*P* > 0.05) included gender, race, asthma, education level, smoking status, body mass index (BMI), alcohol consumption, and hyperlipidemia.

Among continuous variables, the median fasting blood glucose in the heart failure group was 110 mg/dl, compared with 100 mg/dl in the non-heart failure group; the median glycosylated hemoglobin in the heart failure group was 6.1%, vs. 5.6% in the non-heart failure group; the median height of patients in both groups was 170 cm; the median serum albumin in the heart failure group was 41 g/L, compared with 43 g/L in the non-heart failure group; the median alanine aminotransferase in the heart failure group was 20 U/L, vs. 24 U/L in the non-heart failure group; the median serum osmolality of patients in both groups was 280 mmol/kg; the median serum total calcium in the heart failure group was 2.3 mmol/L, compared with 2.4 mmol/L in the non-heart failure group; the median serum potassium in the heart failure group was 4.2 mmol/L, vs. 4.0 mmol/L in the non-heart failure group; the median serum sodium of patients in both groups was 140 mmol/L; the median serum glucose in the heart failure group was 6.2 mmol/L, compared with 5.4 mmol/L in the non-heart failure group; the median serum iron in the heart failure group was 14 μmol/L, vs. 15 μmol/L in the non-heart failure group; the median sedentary time in the heart failure group was 450 min, compared with 360 min in the non-heart failure group; the median neutrophil-to-platelet activation ratio (NPAR) in the heart failure group was 150, vs. 130 in the non-heart failure group; the median neutrophil-to-lymphocyte ratio (NLR) in the heart failure group was 2.4, compared with 1.9 in the non-heart failure group; and the median systemic immune-inflammation response index (SIRI) in the heart failure group was 1.5, vs. 0.98 in the non-heart failure group. Variables not significantly associated with heart failure (*P* > 0.05) included waist circumference, serum globulin, aspartate transaminase, γ-glutamyl transferase, serum bicarbonate, and systemic immune-inflammation index (SII).

### Model feature selection

**Figure 2:**
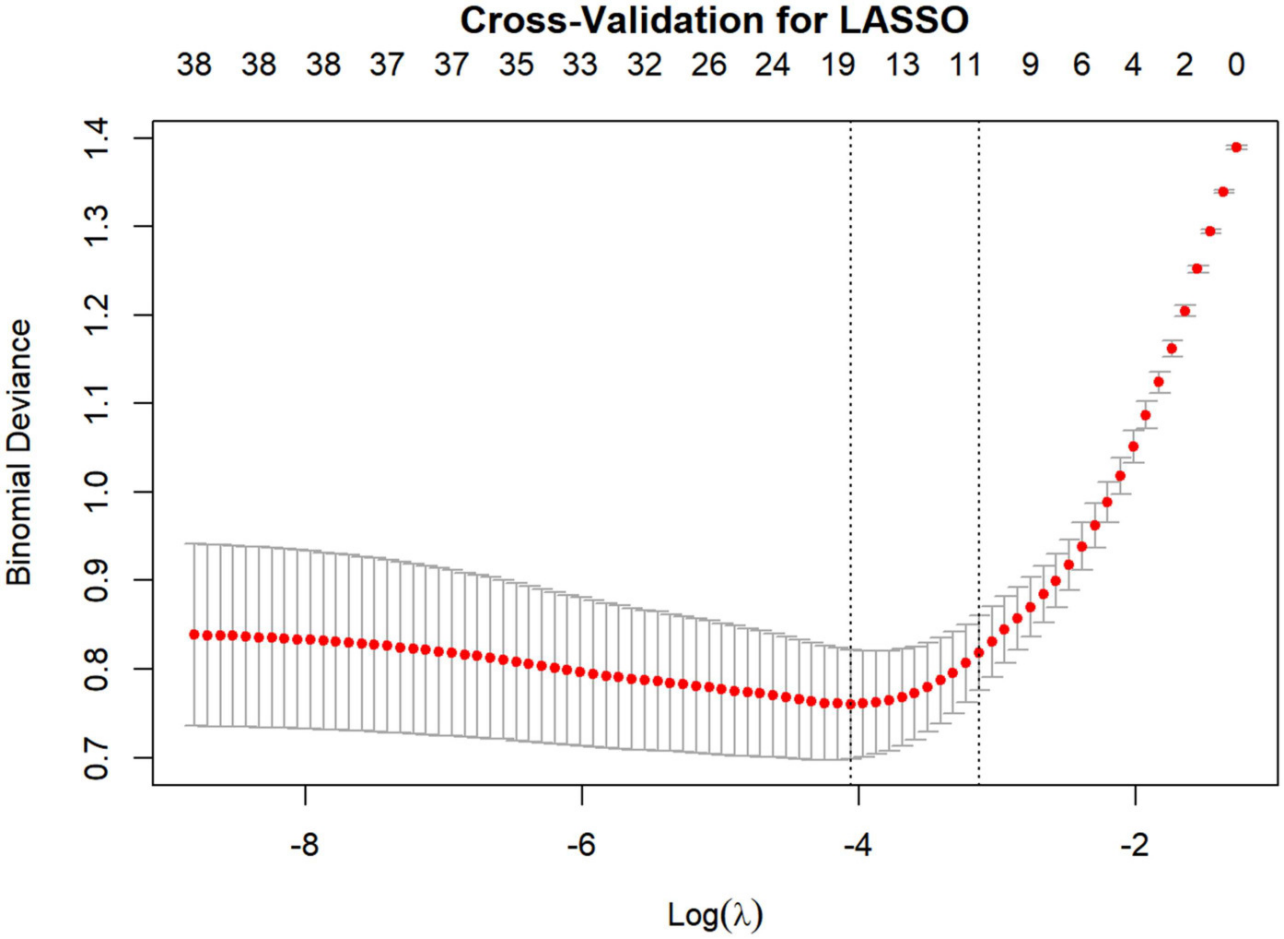
LASSO cross - validation: binomial deviance vs. Log(*λ*).

**Figure 3:**
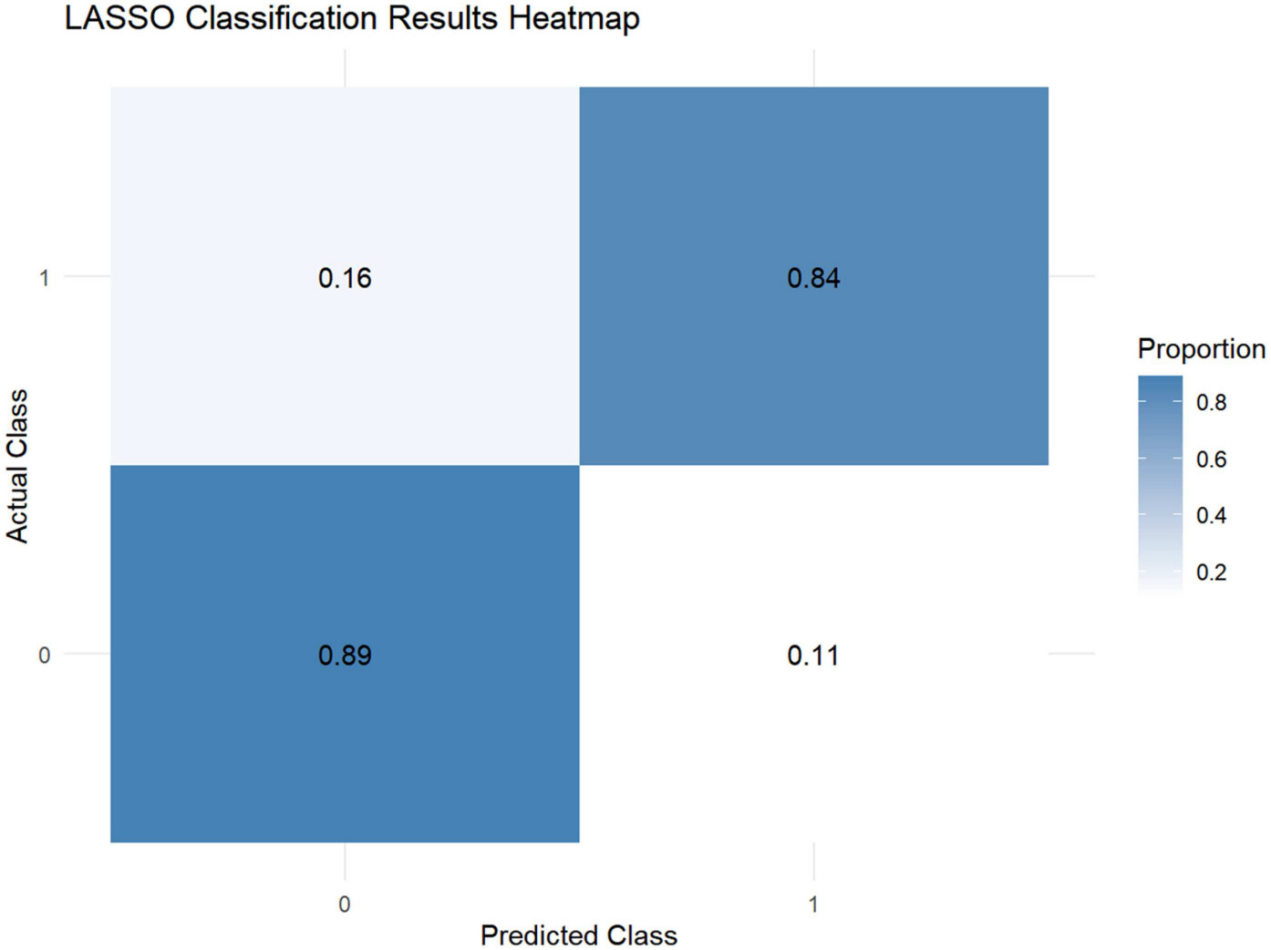
Heatmap of LASSO classification performance.

**Figure 4:**
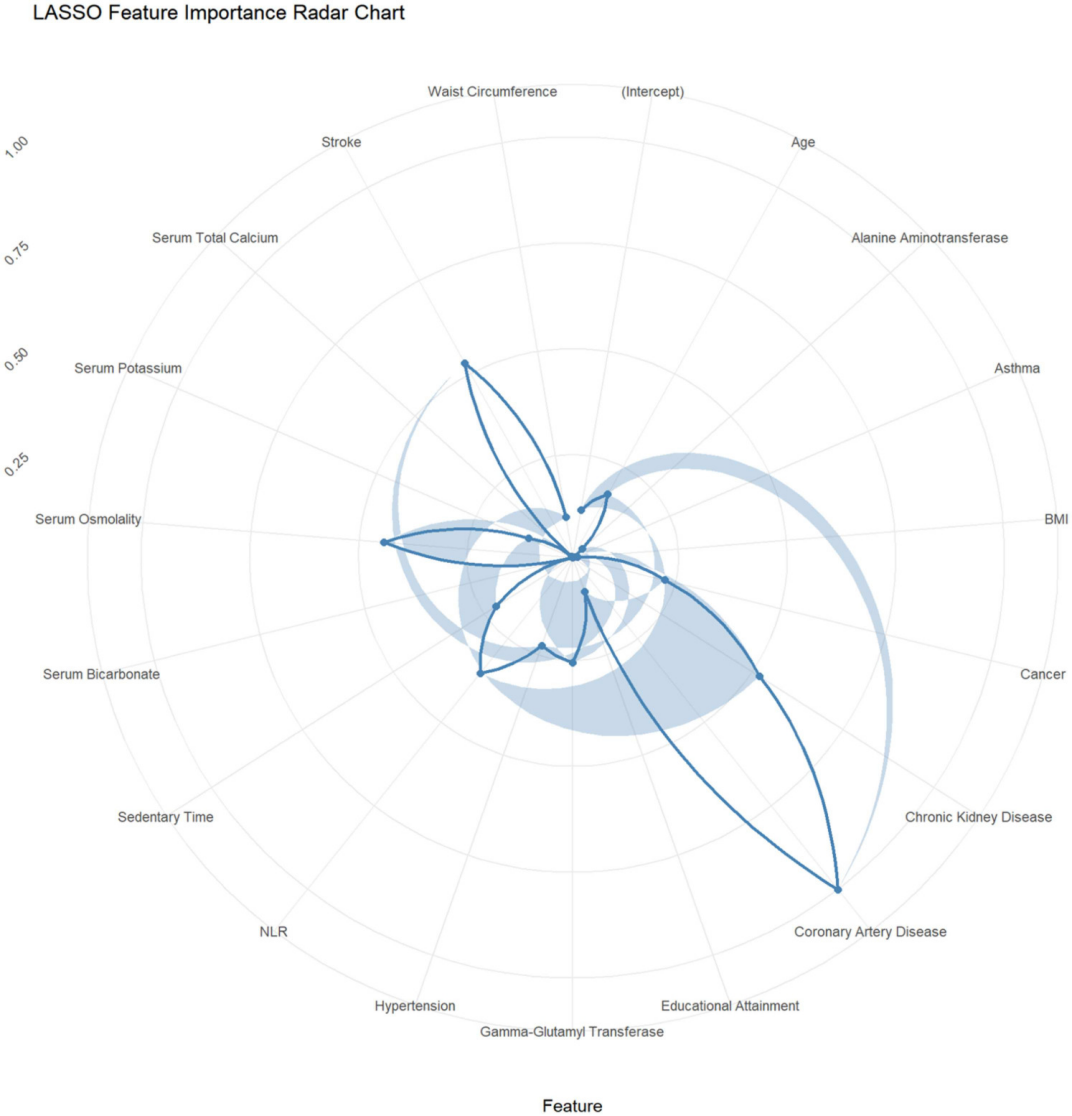
Radar chart of feature importance from LASSO regression.

**Figure 5:**
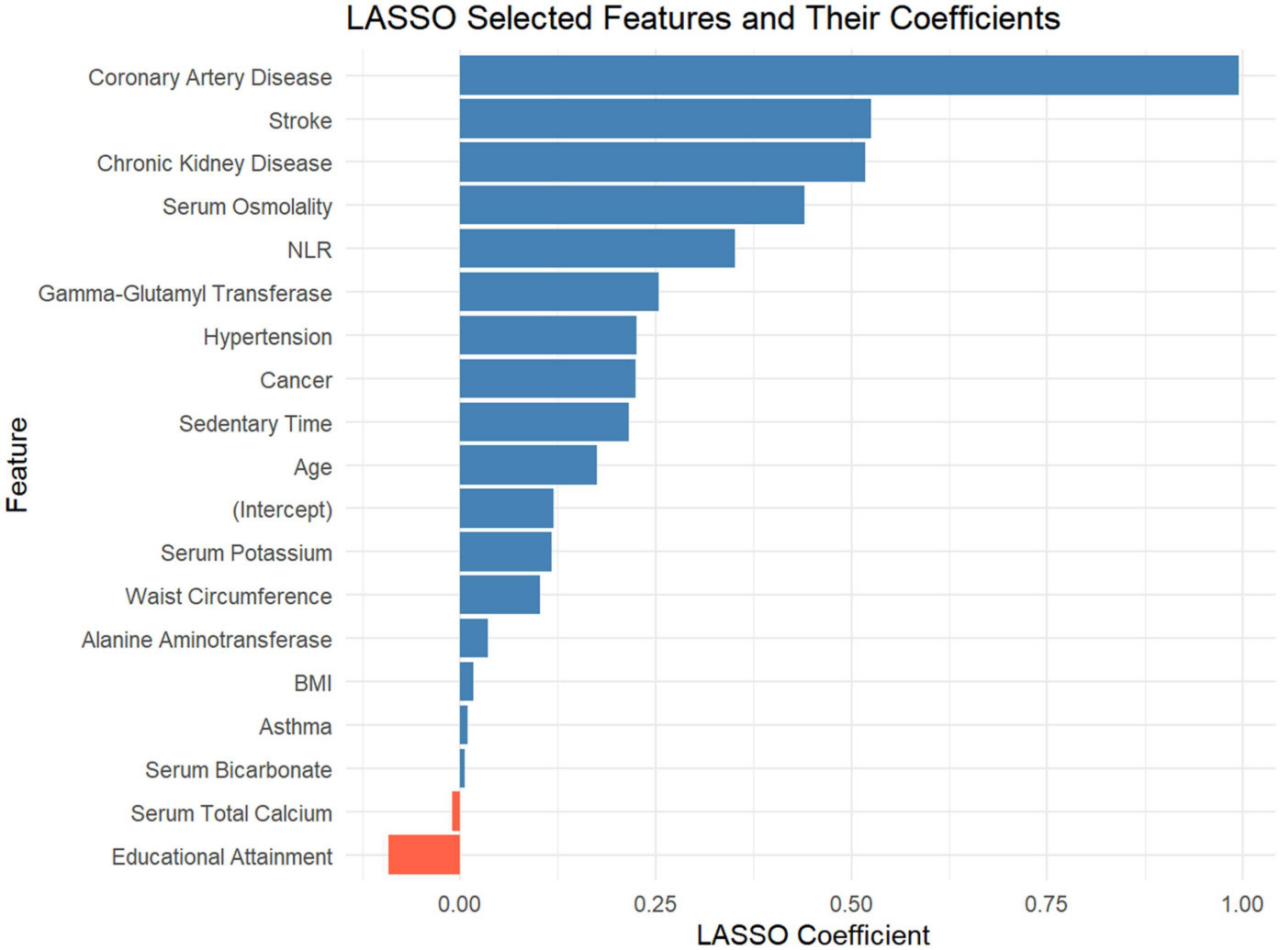
LASSO regression: selected features and coefficient magnitudes.

This study employed the LASSO regression model for feature selection, aiming to identify key factors closely associated with the development and progression of heart failure in patients with hyperuricemia from multidimensional indicators. By plotting the LASSO cross-validation curve (Cross-Validation for LASSO), the optimal regularization parameter was determined based on the trend of binomial deviance with log-transformed penalty coefficients [Log(*λ*)]. This effectively balanced model complexity and predictive performance, preventing overfitting (Figure 2). The LASSO Classification Results Heatmap intuitively demonstrates the model's classification efficiency; the proportion of overlapping areas between actual and predicted categories reflects classification accuracy, verifying the clinical application value of the selected features (Figure 3). The LASSO Feature Importance Radar Chart (Figure 4) and LASSO Selected Features and Their Coefficients (Figure 5) further quantify the impact intensity of key features (such as Coronary Artery Disease and Stroke) on heart failure, providing new perspectives for research on disease mechanisms. The screened key features were incorporated into subsequent machine learning models, offering a basis for precise diagnosis and treatment of hyperuricemia complicated with heart failure and promising to advance clinical research and practice in this field.

### Model performance evaluation

**Figure 6:**
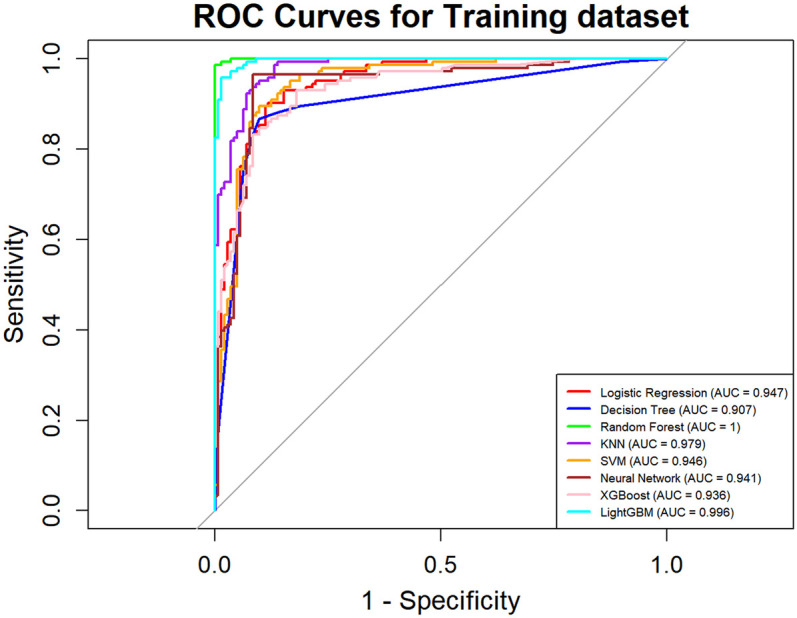
ROC curves of machine learning models on training dataset for heart failure prediction.

**Figure 7:**
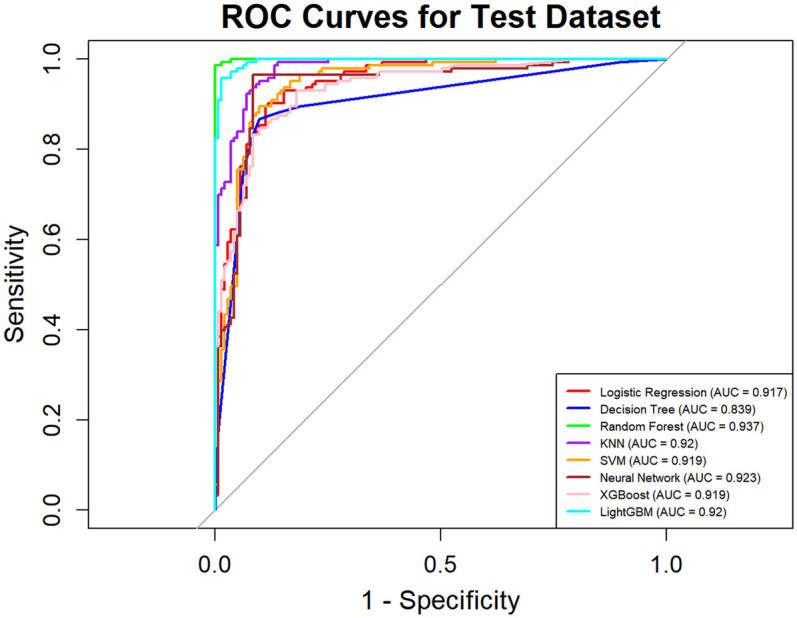
ROC curves of machine learning models on test dataset for heart failure prediction.

**Figure 8:**
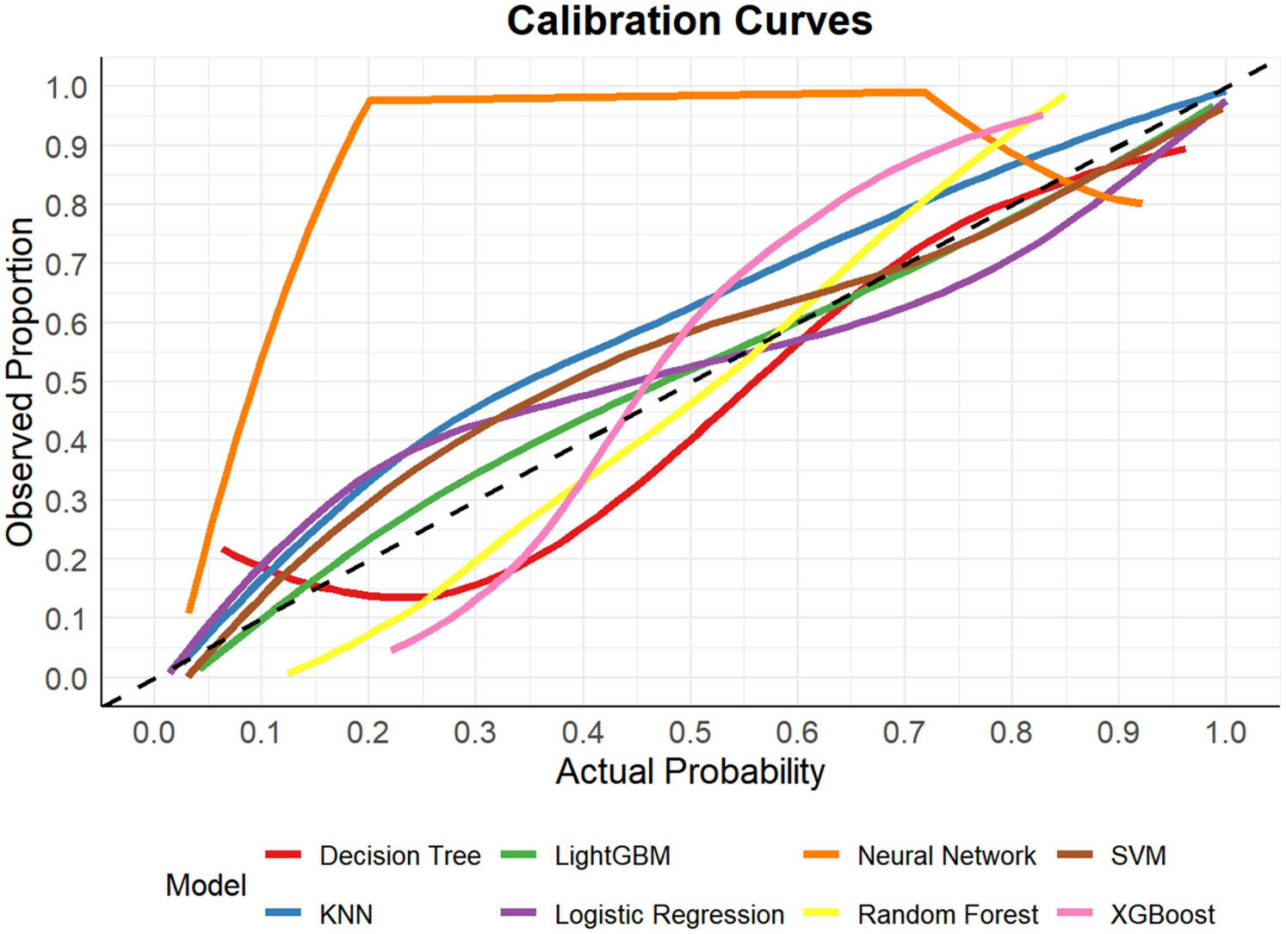
Calibration curves of machine learning models for heart failure risk prediction.

**Figure 9:**
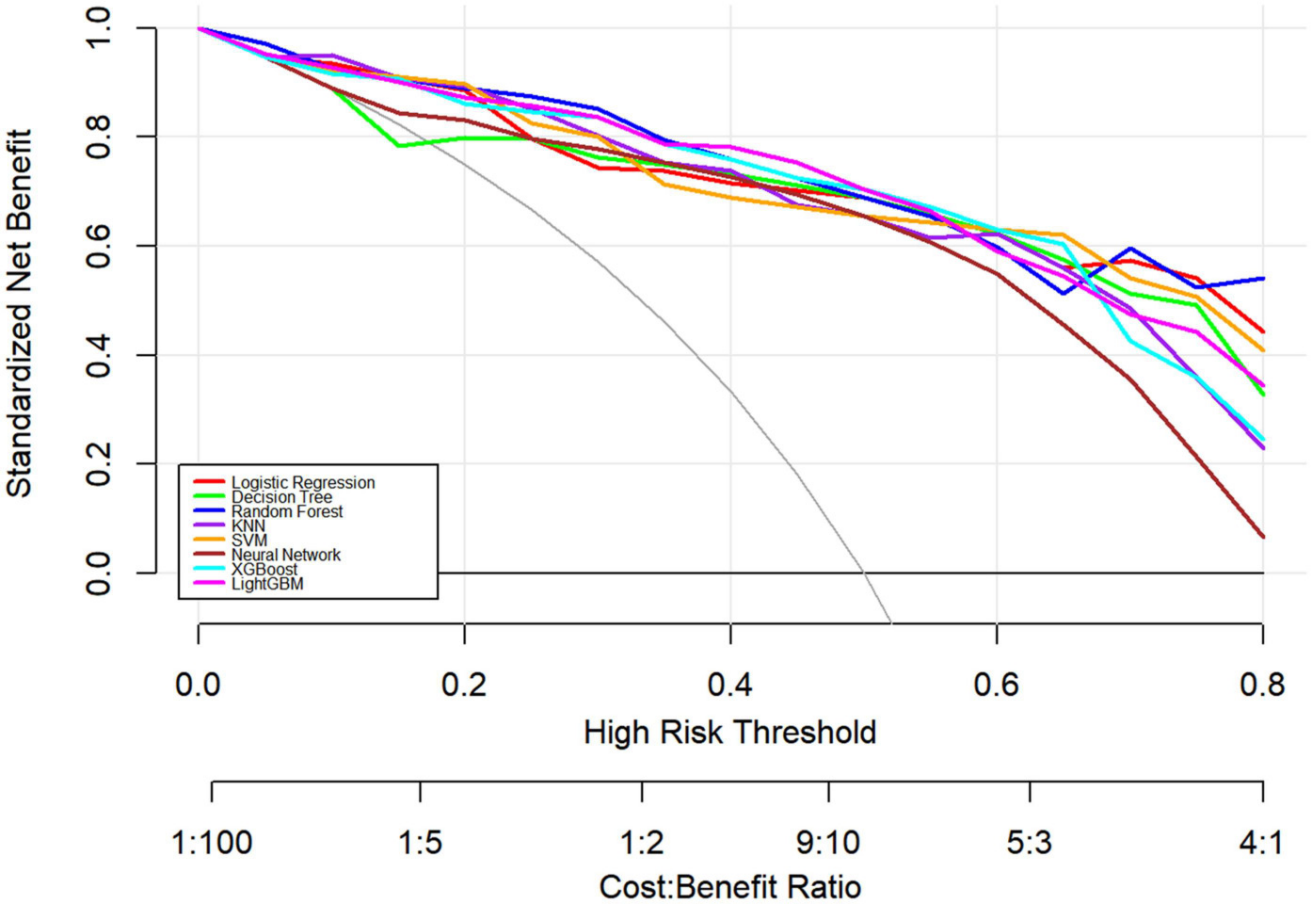
Decision curve analysis of machine learning models for heart failure risk stratification.

In this study, a total of 8 machine learning models—Logistic Regression, KNN, Decision Tree, Random Forest, XGBoost, LightGBM, SVM, and Neural Network—were constructed for predicting heart failure risk in patients with hyperuricemia. Performance evaluation was conducted from multiple dimensions, including basic indicators such as accuracy and sensitivity (Table 2), combined with ROC curves (Figures 6, 7), calibration curves (Figure 8), decision curve analysis (DCA) (Figure 9), and the Hosmer-Lemeshow (H-L) test (Table 3), providing a basis for clinically screening the optimal tool.

**Table 2:** Performance metrics of machine learning models for heart failure risk prediction in hyperuricemia patients.

Model	Dataset	Accuracy	Sensitivity	Specificity	Positive predictive value	Negative predictive value	F1 score
Logistic	Train	0.8776	0.8671	0.8881	0.8857	0.8699	0.8763
Logistic	Test	0.8443	0.8361	0.8525	0.85	0.8387	0.843
KNN	Train	0.9126	0.8881	0.9371	0.9338	0.8933	0.9104
KNN	Test	0.8443	0.8197	0.8689	0.8621	0.8281	0.8403
Decision tree	Train	0.8846	0.8671	0.9021	0.8986	0.8716	0.8826
Decision tree	Test	0.8443	0.8197	0.8689	0.8621	0.8281	0.8403
Random forest	Train	0.979	0.993	0.965	0.966	0.9928	0.9793
Random forest	Test	0.8443	0.8852	0.8033	0.8182	0.875	0.8504
XGBoost	Train	0.8706	0.8671	0.8741	0.8732	0.8681	0.8702
XGBoost	Test	0.8525	0.8852	0.8197	0.8308	0.8772	0.8571
LightGBM	Train	0.9615	0.972	0.951	0.9521	0.9714	0.9619
LightGBM	Test	0.8607	0.9016	0.8197	0.8333	0.8929	0.8661
SVM	Train	0.8951	0.8881	0.9021	0.9007	0.8897	0.8944
SVM	Test	0.8279	0.8033	0.8525	0.8448	0.8125	0.8235
Neural network	Train	0.9406	0.965	0.9161	0.92	0.9632	0.942
Neural network	Test	0.8279	0.8689	0.7869	0.803	0.8571	0.8346

**Table 3:** Hosmer-Lemeshow test results of machine learning models across datasets.

Model	Dataset	Hosmer - Lemeshow chi - square value	Degrees of freedom	*P*-value
Logistic	Training set	0.6564	1	0.4178
KNN	Training set	10.3777	1	0.0013
Decision tree	Training set	<0.0001	1	1
Random forest	Training set	22.223	1	<0.0001
XGBoost	Training set	20.4673	1	<0.0001
LightGBM	Training set	12.4102	1	0.0004
SVM	Training set	1.8217	1	0.1771
Neural network	Training set	0.0409	1	0.8398
Logistic	Test set	5.5967	1	0.018
KNN	Test set	2.7708	1	0.096
Decision tree	Test set	1.0009	1	0.3171
Random forest	Test set	5.4061	1	0.0201
XGBoost	Test set	6.5828	1	0.0103
LightGBM	Test set	0.2385	1	0.6253
SVM	Test set	0.9476	1	0.3303
Neural network	Test set	17.8888	1	<0.0001

Screened by the Hosmer-Lemeshow (H-L) test, only the Decision Tree (training set *P* = 1.0000, test set *P* = 0.3171) and SVM (training set *P* = 0.1771, test set *P* = 0.3303) showed *P*-values >0.05 in both the training and test sets, thus proceeding to subsequent comparisons. A comparison of their performance revealed the following: in the ROC curves, the AUC of SVM in the training set (0.946) was higher than that of the Decision Tree (0.907), and the AUC of SVM in the test set (0.919) was also higher than that of the Decision Tree (0.839). In terms of basic indicators, the training set SVM exhibited good performance, with an accuracy of 0.8951 and a sensitivity of 0.8881, enabling effective identification of high-risk individuals; although the accuracy of the test set SVM was 0.8279, slightly lower than that of the Decision Tree, its sensitivity (0.8033) was comparable, maintaining the ability to identify positive samples. Calibration curve analysis showed that the predicted probabilities of SVM had a good fit with the actual observed probabilities, indicating high reliability of its output probabilities. Decision curve analysis (DCA) further verified that SVM achieved a higher standardized net benefit in most high-risk threshold intervals. In summary, the SVM model performed better in predicting heart failure risk in patients with hyperuricemia and can provide effective support for clinical risk assessment. Therefore, the SVM model was selected for subsequent analysis.

### Shapley additive exPlanations analysis

To further explore the mechanism by which the Support Vector Machine (SVM) model predicts heart failure risk in patients with hyperuricemia, this study employed SHapley Additive exPlanations (SHAP) values. SHAP values can accurately dissect the contribution of each feature to the model's prediction results, aiding in identifying key predictors and their directions of action in risk stratification for heart failure in patients with hyperuricemia.

**Figure 10:**
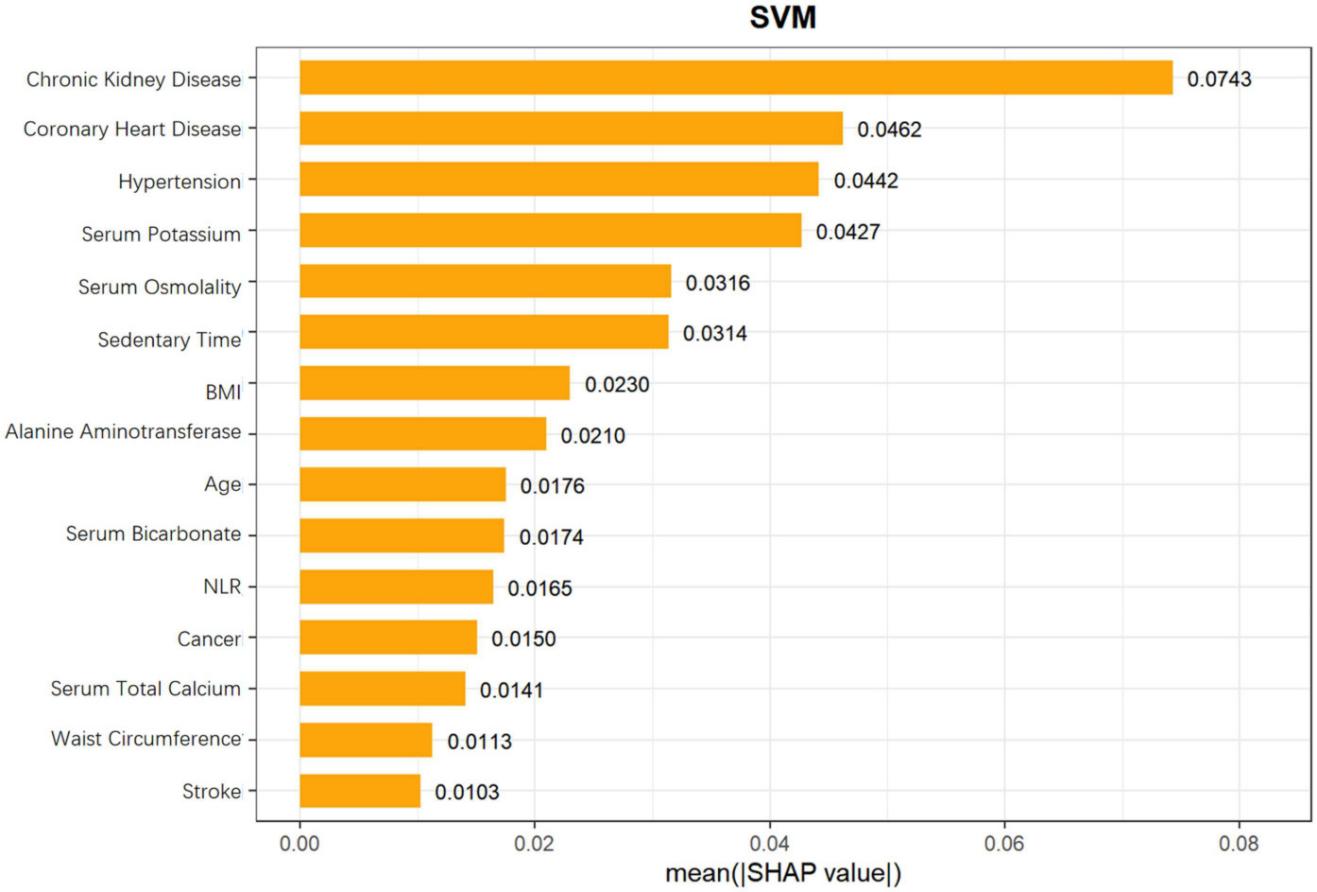
Mean SHAP values of features in SVM model for heart failure risk prediction in hyperuricemia patients.

**Figure 11:**
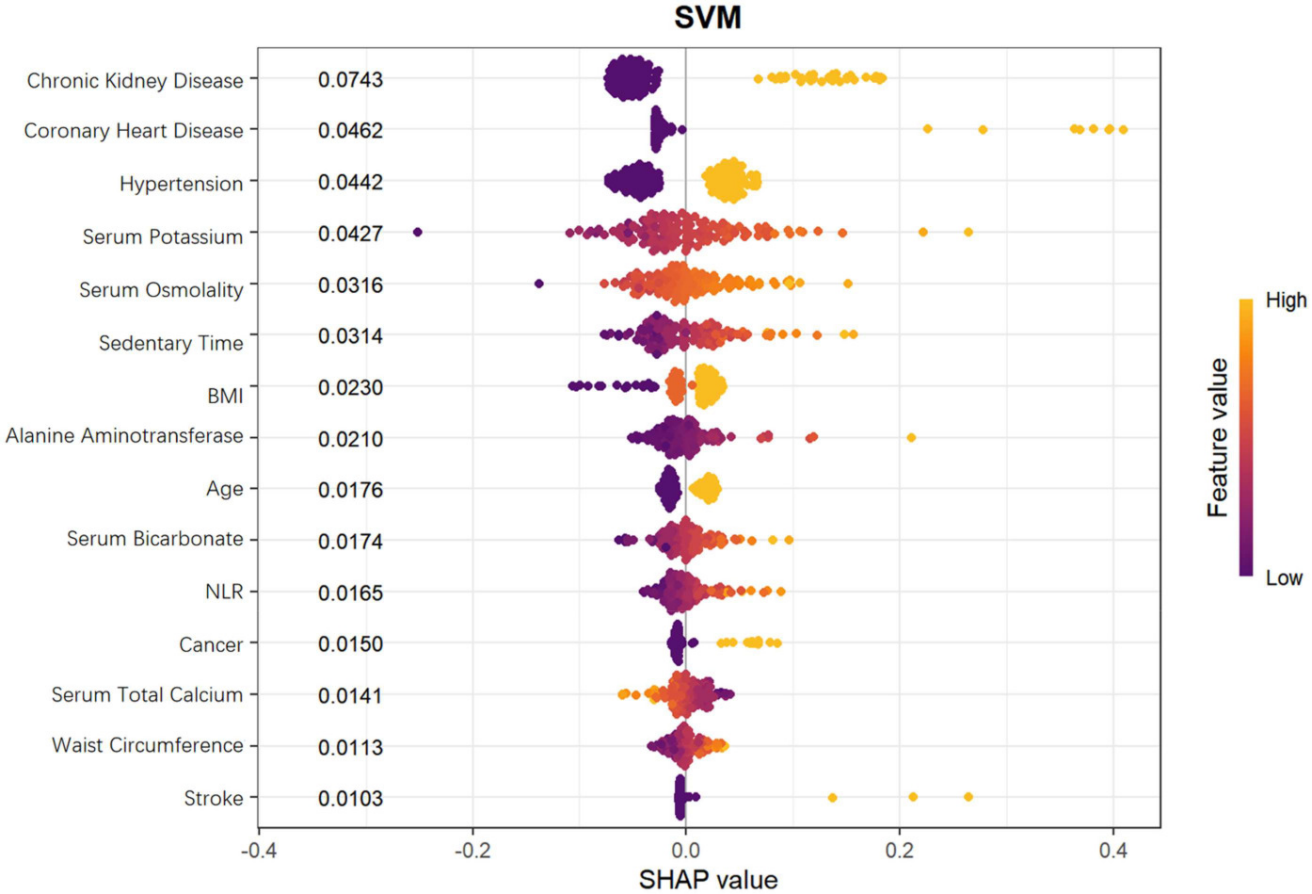
SHAP value distribution of features in SVM model for heart failure risk stratification.

From the SHAP feature importance bar chart (Figure 10), the top six key influencing factors, in order, were chronic kidney disease, coronary heart disease, hypertension, serum potassium, serum osmolality, and sedentary time, with their average SHAP contribution values reaching 0.0743, 0.0462, 0.0442, 0.0427, 0.0316, and 0.0314, respectively. Individuals with high SHAP values mainly provided negative contributions, while those with low SHAP values provided positive contributions, resulting in a specific risk-related trend in the distribution of SHAP values. Further analysis using SHAP scatter plots (Figure 11) revealed that the distribution of feature values for chronic kidney disease, coronary heart disease, hypertension, serum potassium, serum osmolality, and sedentary time was significantly associated with SHAP values: low feature values mostly corresponded to negative SHAP values, while the proportion of positive SHAP values increased with high feature values, generating positive SHAP values. This suggests a bidirectional regulatory effect on heart failure risk prediction, where high values may enhance the risk prediction signal, indicating that these factors are associated with increased risk.

SHAP analysis identified the top 6 key factors and their mechanisms in the SVM model, supporting research on hyperuricemia patients' HF risk prediction and clinical assessment improvement. Their significance indicates application potential in disease risk assessment, aiding comprehensive health evaluation and personalized interventions.

## Discussion

This study analyzed NHANES hyperuricemia data to explore multidimensional indicators' value in predicting HF risk. From routine clinical tests, they offer a convenient tool for cardiovascular risk prediction.

This study found that chronic kidney disease, coronary heart disease, hypertension, serum potassium, serum osmolality, and sedentary time were significantly associated with HF risk in hyperuricemia patients. While CKD, CHD, and hypertension are established HF risk factors routinely considered in clinical practice and incorporated in traditional risk scores (e.g., Framingham, MAGGIC), our study confirms their relevance specifically in hyperuricemia patients, providing population-specific evidence. Moreover, the integration of these established risk factors with additional, readily measurable markers—serum potassium, serum osmolality, and sedentary time—using an interpretable machine learning framework offers quantitative insights into feature importance via SHAP values, which is not captured by conventional scoring systems, and may improve individualized risk stratification in this specific population.

Furthermore, our findings can be compared with recent NHANES-based machine learning studies, in which LightGBM and SHAP were applied to evaluate the association between the visceral fat metabolism score (METS-VF) and heart failure prevalence in U.S. adults, indicating that composite metabolic-adipose biomarkers may outperform traditional risk factors such as hypertension and coronary heart disease in HF prediction. Similar to our approach, SHAP analysis provided interpretable rankings of feature importance, highlighting the value of integrating machine learning with explainable AI frameworks. This comparison emphasizes that multidimensional indicators—including metabolic, renal, and lifestyle factors—can be effectively leveraged through ML-SHAP approaches to enhance HF risk stratification, supporting the broader applicability of our interpretable ML framework across diverse clinical settings (17).

This study found chronic kidney disease significantly predicts HF risk in hyperuricemia patients, consistent with literature. Hyperuricemia may damage renal tubules, accelerate CKD progression, and raise mortality risk. Increased xanthine oxidase activity (a key cause of hyperuricemia) promotes oxidative stress and inflammatory cytokines, inducing cardiac fibrosis and left ventricular dysfunction. Hyperuricemia and CKD (alone or together) increase HF patients' death risk, emphasizing their joint management in HF treatment (18–21).

Elevated serum potassium levels are associated with reduced renal ion excretion and the use of medications to slow CKD progression. Among these, CKD is the most common cause of elevated potassium levels (22–24). Hyperkalemia exerts harmful effects on cardiac electrophysiology, subsequently inducing malignant arrhythmias and sudden cardiac death, and is positively correlated with the risk of death in patients with cardiovascular disease. However, we do not claim a direct mechanistic link; rather, we emphasize that elevated serum potassium serves as a known risk marker associated with HF in this population. Correcting hyperkalemia may delay the onset and progression of cardiovascular disease (CVD) by regulating oxidative stress, inflammation, and vascular remodeling (25–27). Thus, hyperuricemia itself can indirectly increase the risk of elevated serum potassium by impairing renal tubular function and accelerating CKD progression; when hyperuricemia coexists with hyperkalemia, their combined damage to the heart may exhibit a significant synergistic effect. Hyperuricemia impairs cardiac function by inducing oxidative stress, inflammatory responses, and myocardial fibrosis, while hyperkalemia triggers arrhythmias by disrupting myocardial electrophysiological stability. These two pathological mechanisms interact, collectively exacerbating cardiac dysfunction and ultimately promoting the development and progression of HF.

Elevated serum osmolality activates metabolic processes (e.g., antidiuretic hormone release, aldose reductase-fructokinase pathway), potentially linked to renal injury. It correlates closely with CKD development (5 mmol/L change increases risk by 24%) (28–30) and may drive hypertension (5 mOsm/L rise raises risk by 13%) (32–34). It activates immune macrophages/T cells, amplifying inflammation and causing persistent renal vasoconstriction (31–36), making it a CKD risk factor. Its elevation associates with insufficient water intake, high salt (32), and fructose-containing drinks (37). Lifestyle measures (e.g., more water, less salt) may help prevent renal function decline, but we do not infer direct causation between osmolality and HF.

Hyperuricemia is a risk factor for hypertension (38). Its occurrence (due to abnormal uric acid synthesis/excretion) induces endothelial dysfunction, contributing to hypertension, atherosclerosis, etc (39). It links closely to cardiovascular risk: xanthine oxidase produces uric acid and reactive oxygen species (ROS) simultaneously; intracellular uric acid promotes ROS, both regulating signaling pathways, whose alterations may cause atherosclerosis (40). It increases CHD patients' risks of LVH, enlarged LVEDD, reduced LVEF, and HF. Elevated serum UA promotes LVH via UA-activated mechanisms, inducing ROS (e.g., oxygen free radicals), causing endothelial damage, oxidative stress, oxidized LDL, and lipid peroxidation (41).We note these are well-established associations rather than novel mechanistic findings.

Sedentary behavior (waking activities with ≤1.5 METs energy expenditure, e.g., sitting, TV viewing) (42) is a core lifestyle element (43). U.S. adults' daily sedentary time rose from 5.5 h (2007) to 6.4 h (2016), accounting for 55% of waking hours (44), with its health impact increasingly studied (45). It links to cardiovascular diseases (e.g., incident HF) (46, 47), harms cardiometabolic health, and raises morbidity/mortality (48, 49). Longer sedentary time correlates with higher HF incidence in men ≥45 (50); HF patients' prolonged sedentary behavior worsens disability and mortality (51–54). Sedentary behavior increases hyperuricemia risk (55–57) by elevating proinflammatory cytokines (IL-6, CRP, TNF-α) and reducing anti-inflammatory markers (IL-RA) (58–60). We avoid asserting that sedentary behavior directly causes HF via hyperuricemia; rather, it is confirmed as an associated risk factor, and our ML model quantifies its predictive contribution.

Importantly, in our model, CKD and elevated serum osmolality emerged as core predictors of heart failure risk. Although our study did not directly measure vascular function, evidence from prior studies suggests that CKD is associated with detectable changes in arterial stiffness, endothelial function, and vascular compliance, which are closely linked to cardiovascular outcomes. These systemic vascular alterations may increase cardiac afterload and promote left ventricular remodeling. Regarding serum osmolality, direct clinical evidence linking it to vascular stiffness in humans is limited. However, mechanistic studies indicate that elevated extracellular sodium, a major determinant of serum osmolality, can stimulate endothelial inflammatory signaling, including upregulation of adhesion molecules (VCAM-1, E-selectin) and chemoattractants (MCP-1), promote leukocyte adhesion and transmigration, and accelerate atherosclerotic changes in animal models. These findings suggest that serum osmolality may influence vascular tone, endothelial activation, and hemodynamic stress, which could plausibly contribute to heart failure risk in hyperuricemia patients. Taken together, these observations provide a broader pathophysiological context for our findings, suggesting that routine markers such as CKD status and serum osmolality may serve as proxies for underlying vascular dysfunction, though the precise mechanisms remain to be confirmed in future studies (61, 62).

In this study, chronic kidney disease, coronary heart disease, hypertension, serum potassium, serum osmolality, and sedentary time were closely associated with HF occurrence in patients with hyperuricemia, consistent with previous studies and supporting their potential clinical value.

This study's strengths include the use of a nationally representative NHANES dataset, incorporation of multidimensional clinical indicators, and comparison across multiple machine learning algorithms. The SVM model exhibited stable and superior overall performance across discrimination, calibration, and clinical utility metrics, demonstrating its potential value for individualized HF risk assessment among hyperuricemia patients. Although the top predictors were known HF risk factors, the main contribution lies in demonstrating the feasibility and interpretability of ML for risk stratification in this population.

In certain cases, the relatively high AUC (∼0.92) observed for the SVM model may be partly influenced by the limited number of HF events and sample size. To reduce potential overfitting, the dataset was balanced using SMOTEENN, and LASSO logistic regression with 10-fold cross-validation was applied for feature selection. Collinearity among selected features was assessed prior to model training to ensure stability. Selected features included demographic, biochemical, hematological, and inflammatory indices, chosen based on clinical availability, relevance to HF and cardiovascular risk, and prior literature evidence. Although nested cross-validation was not performed, model performance was evaluated on an independent test set, which was not used during feature selection or training, providing some support for model robustness.

It should be noted that a substantial proportion of participants were excluded due to missing values for some clinical, laboratory, and lifestyle variables, which were not consistently available across the entire NHANES survey cycles. This high exclusion rate mainly reflects differences in variable availability across survey years, rather than selective removal of specific participant subgroups. As each excluded participant had a different pattern of missing variables, direct comparison between participants with complete vs. incomplete data was not feasible. Some participants lacked biochemical indicators, while others lacked lifestyle or examination data, and these missing patterns were not uniform across individuals or survey cycles. Despite these limitations, all data were obtained from the official NHANES website, ensuring standardized quality control and data authenticity. Therefore, the analysis remains reliable and valid within the scope of available variables.

However, as NHANES is a cross-sectional dataset, causal relationships between predictors and heart failure cannot be established, and the predictive model lacks time-course or longitudinal validation. Furthermore, heart failure was identified based on self-reported physician diagnosis, which may be subject to recall bias and misclassification, potentially leading to under- or overestimation of associations. Essential diagnostic data, such as BNP levels or echocardiography, were unavailable, and HF subtypes (e.g., HFrEF vs. HFpEF) and severity were not specified. Additionally, the dataset lacks information on participants' medication use (e.g., diuretics, ACE inhibitors, urate-lowering therapy), dietary patterns, and socioeconomic status, which may confound or modify observed associations with heart failure risk. Therefore, the correlations between hyperuricemia and HF in this study should be interpreted as associative rather than mechanistic, and the observed associations should be interpreted with caution. Future prospective cohort studies are warranted to validate the predictive performance of the model over time, examine temporal relationships between risk factors and HF onset, and assess its clinical utility in longitudinal settings.

The number of HF cases is limited, and even with SMOTEENN balancing, there may be a risk of model overfitting and restricted generalizability. Moreover, because the data are primarily from the U.S., the generalizability of our findings to other countries, healthcare settings, or populations may be limited, highlighting the need for validation in more diverse cohorts. Additionally, bootstrapped confidence intervals for model performance were not calculated, which could be addressed in future studies to further quantify predictive uncertainty.

Therefore, the results should be considered exploratory. Nevertheless, this study provides valuable preliminary evidence supporting the feasibility of constructing a simple, interpretable machine learning framework based on readily available clinical indicators. SHAP analysis enhances model interpretability, helping to understand the contribution of key features to HF risk and providing a foundation for future longitudinal studies and multicenter validation, while avoiding unsupported causal claims.

In terms of clinical applicability, the proposed SVM-based model uses routinely available and low-cost clinical parameters—such as blood pressure, serum electrolytes, and basic metabolic indicators—which are widely obtainable in both primary care and cardiology settings. This characteristic facilitates its potential integration into existing electronic health record (EHR) systems for automated risk flagging during routine visits. In primary care, the model could assist general practitioners in identifying hyperuricemia patients who require early cardiovascular evaluation or referral, while in cardiology clinics, it may support comprehensive HF risk stratification and follow-up planning.

However, several barriers to implementation should be acknowledged. First, because the model was developed using U.S. NHANES data, its generalizability to other healthcare systems or ethnic groups remains uncertain, necessitating local recalibration and validation before clinical deployment. Second, the model currently requires basic computational infrastructure and trained personnel to interpret SHAP outputs, which may limit adoption in resource-limited primary care settings. Third, as the present model is research-based and not yet integrated into clinical software, further translational studies are needed to assess its real-world performance, workflow compatibility, and impact on patient outcomes. Addressing these barriers through external validation, user-friendly interface design, and clinician education will be essential for successful implementation in everyday practice.

This research developed a simple tool for predicting HF risk in hyperuricemia patients. Integrating it into clinics improves high-risk detection, supports early intervention, and optimizes outcomes.

## Conclusion

In summary, the present study identifies chronic kidney disease, coronary heart disease, hypertension, serum potassium concentrations, serum osmolality, and sedentary time as the most influential indicators associated with heart failure among individuals with hyperuricemia. While CKD, CHD, and hypertension are well-established HF risk factors already incorporated into traditional risk scores, our model provides added value by integrating these with additional, easily obtained variables—serum potassium, serum osmolality, and sedentary time—within an interpretable machine learning framework that quantifies their relative contributions to HF risk. However, due to the cross-sectional nature of NHANES, the model lacks time-course validation. Future longitudinal and prospective studies are necessary to confirm these associations and evaluate the predictive performance of the model over time. Although these findings highlight the potential of interpretable machine learning for early HF risk identification in hyperuricemia, they should be regarded as exploratory due to the cross-sectional nature of the dataset and the relatively small number of HF cases. Future prospective studies and external validations in more diverse populations are warranted to confirm the predictive value of these factors and to assess the generalizability and clinical utility of the proposed model.

From a practical perspective, the model's reliance on routinely collected, low-cost parameters enhances its potential for real-world use. With appropriate external validation and digital integration, it could be implemented as a clinical decision-support tool in primary care to guide early cardiovascular screening among hyperuricemia patients, or in cardiology to complement existing HF risk assessments. Nonetheless, barriers such as data system compatibility, model interpretability training, and local calibration requirements must be addressed before widespread clinical adoption.

Despite these limitations, this study provides a valuable foundation for future research on cardiovascular risk prediction in hyperuricemia and underscores the promise of integrating machine learning interpretability into clinical decision support systems.

## Data Availability

Publicly available datasets were analyzed in this study. This data can be found here: https://wwwn.cdc.gov/nchs/nhanes/Default.aspx.
